# Allelic imbalance in 1p, 7q, 9p, 11p, 12q and 16q regions in non-small cell lung carcinoma and its clinical association: a pilot study

**DOI:** 10.1007/s11033-013-2782-1

**Published:** 2013-10-04

**Authors:** Karolina H. Czarnecka, Monika Migdalska-Sęk, Adam Antczak, Dorota Pastuszak-Lewandoska, Jacek Kordiak, Ewa Nawrot, Daria Domańska, Dorota Kaleta, Paweł Górski, Ewa Barbara Brzeziańska

**Affiliations:** 1 Department of Molecular Bases of Medicine, Medical University of Lodz, Pomorska Str. 251, 92-213 Łódź, Poland; 2 Department of General and Oncological Pneumology, Medical University of Lodz, Kopcińskiego 22, 90-153 Łódź, Poland; 3 Department of Thoracic Surgery, General and Oncologic Surgery, Medical University of Lodz, Żeromskiego 113, 90-710 Łódź, Poland; 4 Department of Preventive Medicine, Medical University of Lodz, Żeligowskiego 7/9, 90-643 Łódź, Poland; 5 Department of Pneumology and Allergology, Medical University of Lodz, Kopcińskiego 22, 90-153 Łódź, Poland

**Keywords:** Non-small cell lung carcinoma (NSCLC), Loss of heterozygosity (LOH), Microsatellite instability (MSI), Microsatellite markers, Genetic instability

## Abstract

In lung cancer pathogenesis, genetic instability, i.e., loss of heterozygosity (LOH) and microsatellite instability (MSI) is a frequent molecular event, occurring at an early stage of cancerogenesis. The presence of LOH/MSI in non-small cell lung carcinoma (NSCLC) was found in many chromosomal regions, but exclusive of 3p their diagnostic value remains controversial. In this study we focused on other than 3p regions—1p31.2, 7q32.2, 9p21.3, 11p15.5, 12q23.2 and 16q22—the *loci* of many oncogenes and tumour suppressor genes. To analyze the potential role of LOH/MSI involved in NSCLC pathogenesis we allelotyped a panel of 13 microsatellite markers in a group of 56 cancer specimens. Our data demonstrate the presence of allelic loss for all (13) analyzed markers. Total LOH/MSI frequency in NSCLC was the highest for chromosomal region 11p15.5 (25.84 %), followed by 9p21.3 and 1p31.2 (19.87 and 16.67 % respectively). A statistically significant increase of total LOH/MSI frequency was detected for the 11p15.5 region (*p* = 0.0301; χ^2^ test). The associations of total LOH/MSI frequency: 1) increase in 11p15.5 region (*p* = 0.047; χ^2^ test) and 2) decrease in 7q32.2 region (*p* = 0.037; χ^2^ test) have been statistically significant in AJCC III (American Joint Committee on Cancer Staging). In Fractional Allele Loss (FAL) index analysis, the correlation with cigarette addiction has been statistically significant. The increased amount of cigarettes smoked (pack years) in a lifetime correlates with increasing FAL (*p* = 0.024; Kruskal–Wallis test). These results demonstrate that LOH/MSI alternation in studied chromosomal regions is strongly influenced by tobacco smoking but do not seem to be pivotal NSCLC diagnostic marker with prognostic impact.

## Introduction

Lung cancer is one of the leading causes of cancer mortality and morbidity in most developed countries and the most common cause of death from malignant tumours worldwide in man between the ages of 50 and 70 [1, 2]. According to histological verification, lung cancer is classified into two major groups based on its biology, therapy and prognosis: non-small cell lung carcinoma (NSCLC), representing approximately 75 % of all primary lung cancers and small cell lung carcinomas (SCLC), accounting for about 20 % [3]. The following histological types are present in less than 5 % of cases: adenosquamous carcinoma, carcinoid, bronchial gland carcinoma [4]. The major histological and clinical types of NSCLC are squamous cell carcinoma (SCC); adenocarcinoma (AC) and large cell carcinoma (LCC) [3, 4]. Adenocarcinoma is the most commonly occurring cancer type in nonsmokers [3].

The most important risk factor in lung cancer development is tobacco smoking, including second-hand smoke. In a study based on many different populations, it was documented that the incidence of lung cancer increased in a direct proportion to time and the amount of cigarettes smoked [4–6]. Despite the fact that tobacco smoke contains many carcinogenic substances—responsible for initiation of carcinogenesis—only 30 % of smokers finally develop lung cancer [3, 6, 7]. On the other hand, a significant percentage of people who have never smoked develop lung cancer [3, 7]. Apart from environmental factors, many genetic susceptibilities may be involved in carcinogenesis in the lung and numerous molecular changes are responsible for different histopathological types of lung cancer. So far, many molecular alterations (including mutations, polymorphisms, expression changes due to promoter hypermethylation) in many genes localized in different chromosomal regions have been proposed as leading to NSCLC development [8]. These genes are recognized as important in cell cycle regulation, proliferation, survival and metastasis (e.g. *KRAS, BRAF, TP53, MYC, CCND1, EGFR, ERBB2* or *BCL2*) [9, 10].

According to many studies focused on the molecular background of lung carcinogenesis, genetic instability, presented as chromosomal instability via structure and/or number aberration, has been claimed as an universal biomarker [11–13]. Among genetic instabilities, microsatellite instability (MSI) and the more frequently occurring loss of heterozygosity (LOH), where one of the alleles is missing, have been identified as the initiation event in lung carcinogenesis; they are strongly associated with the patient outcome and harbor clinical implications [13, 14]. It has been documented that the common cause (80 %) of LOHs are partial chromosome losses caused by structural alterations [15]. The chromosomally-specific mechanism of LOH may be often associated with deletion of small sequences within the coding region of the gene or non-coding fragment of the genome (microdeletion) where microsatellite sequences are located [16].

As so far many cytogenetic and molecular studies in NSCLC have shown that LOH affects several regions of chromosomes: 1p, 2p, 2q, 3p, 4q, 5q, 6p, 6q, 7q, 7p, 8p, 9p, 10q, 11p, 13p, 13q, 17p, 18q, 19q, 21q and 22q, and some of the markers located in those areas associate with clinicopathological parameters and prognosis [8, 9, 14, 17–20]. Breakpoints are clustered at various chromosomal sites, including 1p13, 3p13, 15p11-q11, 17p11, and 19q13 [21]. However, the most frequent deletions have been identified in 3p, 5q, 8p, 9p, 11p, 18q and 17p. A particular LOH in 17p13 in the *TP53 locus* has been proposed as a prognostic factor for SCLC [21, 22]. Another frequently altered site in lung cancer, 7p12—the *EGFR* *locus*, has been confirmed as a predictive biomarker [3, 23]. According to comparative genome hybridization (CGH) study results, a predominantly metastatic phenotype excluding frequent deletions at 3p12–p14, 3p21, 4p15–p16, 6q24–pter, 8p22–p23, 10q21-qpter and 21q22 occurred more repeatedly in metastatic tumours [20]. Moreover, it has been documented that LOH incidence is strongly influenced by tobacco smoking [8, 19]. Particularly in LCC—one of the major histological types of NSCLC—smoking-induced lung cancer may be associated with 3q and 8q chromosomal gains [24]. Additionally, LOH in 3p21, 9p21, 17p13 and 5q11 has been frequently observed in the bronchial epithelia of smokers, and is significantly associated with the incidence of carcinoma in situ [5, 8, 12, 13].

Despite many molecular studies focused on LOH/MSI alteration in various chromosomal regions in lung cancer, there are still many chromosomal areas potentially harboring some important genes, which have not yet been considered. Those potential candidate regions may have prognostic impact. Taking into account that lung cancer—mainly NSCLC—is often detected too late (only 16 % in localized stage: I, IIA and IIB; recommended to surgical resection) [3, 25] and that among other reasons, lung cancer is still the leading cause of cancer-related death in the world, further studies on molecular biomarkers should be continuously developed.

The *loci* chosen in our study harbor genes (*ARHI, MEST, p16INK4A, KCNQ1, SLC5A8* and *CDHs*) acknowledged as important Tumour Suppressor Genes (TSGs) influencing cell growth, proliferation and apoptosis, and participating in the development and progression of many human tumours other than lung cancers (e.g. breast, ovarian, thyroid). The expression of the above mentioned genes is known to be down-regulated by genetic/epigenetic alterations involving CpG methylation and/or deletion [26–28]. According to our knowledge, only two reports focused mainly on the 12q23.2, 16q11–q21 and 16q23-qter chromosomal regions (harboring *SLC5A8,* *CDH1* and *CDH3* gene *loci*) in lung cell lines and small cohort of tumours have been hitherto published [20, 29]. The aim of the study is to assess whether LOH/MSI alteration in these selected regions may have important diagnostic and/or prognostic value in patients with NSCLC. In our study we focus on the following regions: 1p31.2, 7q32.2, 9p21.3, 11p15.5, 12q23.2 and 16q22.1, where the genes *ARHI, MEST, p16INK4A, KCNQ1, SLC5A8, CDH1* and *CDH3* essential for carcinogenesis are located.

## Materials and methods

### Biological material

The procedures used in the study have been approved by the Ethical Committee of the Medical University of Lodz (RNN/140/10/KE). Written consent was received from each patient. The studied biological material (100–150 mg tissue fragments) was derived from patients (37 men and 19 women) who had undergone pulmunectomy or lobectomy at the Department of Thoracic Surgery, General and Oncologic Surgery, Medical University of Lodz, Poland, from July 2010 to June 2012. The studied tissues included 56 non-small cell lung carcinomas specimens and 56 matching macroscopically unchanged lung tissue samples which were obtained from the margins of resection (most distant site from the resected center of the primary lesion). Immediately after resection, the tissue samples were collected in RNAlater^®^ buffer and frozen in −80 °C. The resected NSCLC specimens and paired macroscopically unchanged tissue were histhopathologically evaluated post-operatively. NSCLC was classified using TNM (Tumour Node Metastasis) classification (pTNM) according to the WHO Histological Typing of Lung Tumour and AJCC staging (American Joint Committee on Cancer Staging) according to the IASCLC Staging Project 7th ed. (2010) Cancer [30].

### Characterization of study patients

The study subjects included 56 patients with diagnosed NSCLC. The clinical characteristics of studied patients and histopathological verifications of non-small cell lung carcinoma are shown in Table 1.

**Table 1:** Clinical characteristics of studied patients and histopathological verifications of NSCLC

Clinical and pathological features	n	%
*Mean age*		
65 ± 8.433 (range 47–87)	56	
Men		
65 ± 8.234 (range 47–87)	37	66
Woman		
63 ± 8.717 (range 47–79)	19	34
*Age group*		
<60	14	25
60–70	25	45
>70	17	30
*Histopathological type of NSCLC*		
Squamous cell carcinoma (SCC)	30	54
SCC keratodes	12	21.4
SCC non-keratodes	11	19.6
Adenocarcinoma (AC)	21	37
AC mucosecretans	6	10.7
AC non-mucosecretans	15	26.8
Large cell carcinoma (LCC)	5	9
*pTNM*		
T1	18	32
T2	22	39
T3–4	16	29
*AJCC*		
AJCC IA	13	23
AJCC IB	11	19.4
AJCC IIA	8	14.6
AJCC IIB	6	11
AJCC III A/IIIB	18	32

*pTNM* post-operative Tumour Node Metastasis classification according to the WHO Histological Typing of Lung Tumour, *AJCC* Joint Committee on Cancer Staging according to the IASCLC Staging Project 7th ed. (2010) Cancer [30]

The complete information about smoking habits was available for 53 patients. Three patients were non-smokers, and 50 were smokers or former smokers. Patients were divided into groups according to their smoking habits: time of tobacco addiction and amount of cigarettes smoked—the latter was presented as pack years (PY) and was calculated according to the NCI Dictionary of Cancer Terms (1 PY is equal to smoking 20 cigarettes per day for 1 year) [31], see Table 2.

**Table 2:** Characteristics of tobacco addiction and consumption (in PY) for studied patients with diagnosed NSCLC

Tobacco addiction and consumption	n = 53	%
*The smoking period*		
Smokers	50	94.3
Less than 25 years	9	17
More than 25 years, less than 39	21	39.6
More than 40 years	20	37.7
Non-smokers	3	5.7
Pack years (PY)		
Up to 20 PY	7	14
20 to 29 PY	10	20
30 to 39 PY	11	22
More than 40 PY	22	44

### DNA extraction

The isolation of genomic DNA from NSCLC specimens and it’s matching macroscopically unchanged lung tissue (serving as a reference sample of DNA) were performed using a QIAamp DNA Mini Kit (Qiagen, Germany), according to the manufacturer’s protocol. The quality and quantity of isolated DNA was spectrophotometrically assessed, by measuring the absorbance at a wavelength of 260/280 nm using Eppendorf BioPhotometr™ Plus (Eppendorf, Germany). DNA samples with a 260/280 nm ratio in the range 1.8–2.0 were considered as high quality and used in further analysis.

### Microsatellite analysis

Markers used to perform microsatellite analysis were elected from the NCBI database (http://www.ncbi.nlm.nih.gov/genome/sts/sts) with supplementary mapping information, if necessary, provided in the Cooperative Human Linkage Centre Database (http://www.chlc.org) or the Genome Database (http://www.gdb.org). The 13 microsatellite markers chosen in our study contained polymorphic microsatellite repeats: (T)n, (CA)n, (TTA)n and (TCTA)n. Markers selected for this study were linked to the following chromosomal regions: 1p31.2, 7q32.2, 9p21.3, 11p15.5, 12q23.2 and 16q22.1 where genes involved in carcinogenesis are located. All forward primers were labelled at the 3′end with fluorescent dye: 6-FAM, NED, PET or VIC. The amplification reactions were performed for each patient using DNA from cancerous tissue paired with its matching DNA from macroscopically unchanged margin tissue. Amplifications were carried out using an AmpliTaq Gold^®^ 360 DNA Polymerase Kit (Applied Biosystems, USA) in a Gradient Mastercycler (Eppendorf, Germany). Reactions were conducted in a total volume of 12.5 μl and the reaction mix contained 30–40 ng DNA, 10 × AmpliTaq Gold^®^ 360 buffer (150 mM Tris–HCl, pH 8.3, 500 mM KCl), 360° GC Enhancer, 5 U/μl AmpliTaq Gold^®^ 360 DNA Polymerase, 25 mM MgCl_2_, 10 mM dNTPs, forward and reverse primers (0.5 μM each) and nuclease-free water. The cycle of the amplification reaction included initial denaturation step at 95 °C for 10 min, followed by 30 cycles of amplification with denaturation at 95 °C for 45 s, primer annealing for 30 s at temperature specific for each marker and an elongation step at 72 °C for 1 min. The temperatures of annealing were experimentally established for each pair of primers: in the range of: 47–50 °C (for D7S2519, D7S2544, D11S4088, D11S1318, D12S1041, D12S1727, and D16S3025), 51–59 °C (for D1S2137, D1S368, D7S530, D9S974, D9S1604, and D16S496). The terminal extension step was performed for 45 min at 72 °C. The chromosomal localization (region/gene) of the microsatellite markers and nucleotide sequences of primers used in the study are shown in Table 3.

**Table 3:** The chromosomal localization (region/gene) of the microsatellite markers, marker ID and nucleotide sequences of primers used in the study

Chromosomal localization of the region (gene)	Marker ID	Nucleotide sequence of microsatellite marker (5′–3′)	
1p31.2 (*ARHI*)	D1S2137	F	ACATCTTTGGTTTGGATAGATG
		R	CAAAACTGCACATTTTGCAC
	D1S368	F	GGGCATTGTTTAGGGGTG
		R	TAGTGGGCTTTACGTCTGC
7q32.2 (*MEST*)	D7S2519	F	GGAGGTTAAGATTTACAG
		R	GCTGTGGTGTATCCTGTG
	D7S2544	F	TCCCCAGACCCCATTC
		R	TCCTGTTCATCCTTCATTCC
	D7S530	F	CGTTGCATTTTAGTGGAGCACAG
		R	CAGCAGTAATGAAAGCAAAACACAG
9p21.3 (*p16INK4A*)	D9S974	F	GAGCCTGGTCTGGATCATAA
		R	AAGCTTACAGAACCAGACAG
	D9S1604	F	CCTGGGTCTCCAATTTGTCA
		R	AGCACATGACACTGTGTGTG
11p15.5 (*KCNQ1*)	D11S4088	F	GGGCAGAGGCAGTGGAG
		R	GCATGTTTCGGGGGTG
	D11S1318	F	CCCGTATGGCAACAGG
		R	TGTGCATGTNCATGAGTG
12q23.2 (*SLC5A8*)	D12S1041	F	AACTGTGGAAAAAGGGGAAC
		R	TGCAACAAACCACCATGG
	D12S1727	F	AGTCACCACTGAAAATCCAC
		R	GAGTGAGACCCCGTAAAAA
16q22.1 (*CDH1, CDH3*)	D16S496	F	GAAAGGCTACTTCATAGATGGCAAT
		R	ATAAGCCACTGCGCCCAT
	D16S3025	F	TCCATTGGACTTATAACCATG
		R	AGCTGAGAGACATCTGGG

*F* forward (sense), *R* reverse (antisense)

In reaction with each microsatellite marker, internal controls were used to prevent contamination for biological contamination (contamination of foreign DNA, blank samples with nuclease-free water), and reagent contamination (deionized water instead of AmpliTaq polymerase).

The PCR products were qualitatively analyzed in 2 % agarose gel electrophoresis and after bromide ethidium staining, the presence of amplification product was visualized in UV light. In order to perform the capillary electrophoresis, 0.5 μl of PCR product was mixed with 0.25 μl GS500-LIZ Size Standard and Hi-Di™ Formamide (both reagents Applied Biosystems, USA) up to the final volume of 10 μl. The obtained mixture was denatured for 5 min at 95 °C and subsequently cooled on ice for 3 min. The separation in capillary electrophoresis was conducted on 3130 × l Genetic Analyzer (Applied Biosystems, Hitachi, USA) and the allele detection was assessed using GeneMapper Software v 4.0, according to the manufacturer’s protocol. The informativeness of the studied samples (heterozygosity) was confirmed when two distinct alleles were detected in the reference DNA sample, that is, from unchanged lung tissue from the same patient. Evaluation of LOH/MSI was performed by calculating the ratio of the fluorescence intensity of the alleles originating from unchanged lung tissue sample (N, normal, i.e., control sample) to the fluorescence intensity of the alleles originating from NSCLC sample (T, tumour). For each informative normal-tumour DNA pair (paired N and T samples), an Allelic Imbalance Ratio (AIR) was calculated. The AIR calculation was based on the maximum allele peak heights (fluorescence intensity), and counted as follows (N1:N2)/(T1:T2) (where N1—normal-allele 1; N2—normal-allele 2; T1—tumour allele 1; T2—tumour allele 2) according to the protocol [26]. LOH in tumour samples was considered indicative when AIR was less than 0.67 or greater than 1.35 (according to the criteria of GeneMapper Software v 4.0). MSI in tumour DNA was considered indicative if one or more additional alleles were present in the tumour DNA sample, as compared with the control DNA sample. LOH/MSI frequency (%) was calculated as a percentage of the presence or absence of LOH/MSI alterations in relation to all informative loci (heterozygous DNA). For the analysis of LOH/MSI frequency in all studied regions and markers according to the tumour characteristics (NSCLC histopathological type and TNM, AJCC staging), LOH/MSI frequency was calculated in relation to all studied specimens. For each sample of NSCLC, the Fractional Allele Loss (FAL) index was also calculated. The FAL index describes the ratio of total number of chromosomal *loci* with LOH or MSI to the total number of informative *loci* in all examined 13 microsatellite *loci*.

### Statistical analysis

The obtained results were subjected to statistical analysis with calculation of statistical means, medians and standard deviations. In order to identify the possible association between clinical variables of patients (age at diagnosis, smoking habits), characteristics of the tumour (e.g., tumour size, according to TNM classification, tumour preoperative and postoperative staging according to AJCC) and FAL index values, a Kruskal–Wallis correlation coefficient test was performed. For an analysis of the association between patient’s sex and FAL index values Mann–Whitney’s correlation coefficient test was performed. For comparisons of FAL index values among different chromosomal regions and markers, Fisher’s test was used. In order to analyse the possible association between total LOH/MSI frequency and chromosomal regions or markers, the *χ* ^2^ test was performed. Statistical significance was determined at the level of *p* < *0.05*. The results are presented as mean or median ± SEM and ± SD values. For calculations, Statistica for Windows v. 10 program was applied.

## Results

### Allelic imbalance and microsatellite instability

Paired DNA specimens (DNA from cancerous tissue paired with it’s matching DNA from macroscopically unchanged margin tissue) isolated from all 56 patients were submitted to the LOH/MSI analyses. A panel of 13 microsatellite markers was used in the analysis. The whole of the studied DNA (56 samples) derived from NSCLC specimens was informative at least for two studied *loci*. LOH/MSI changes were observed in all (13/13—100 %) microsatellite markers.

**Fig. 1:**
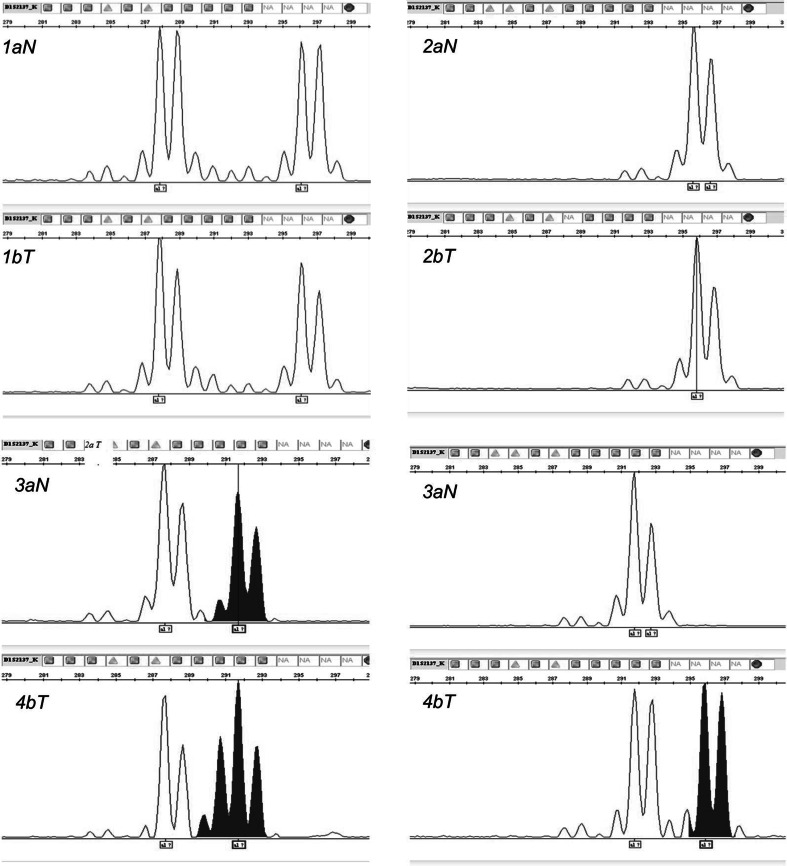
LOH/MSI analysis in NSCLC specimens—an example for LOH/MSI analysis of the D1S2137 marker (3130xl Genetic Analyzer, GeneMapper Software v. 4.0; Applied Biosystems, Hitachi) *Note* *T* tumour sample from NSCLC specimen, *N* control from macroscopically unchanged lung tissue from the same patient. *1a* N—heterozygous DNA/*1b* T—heterozygous DNA (sample no 2); *2a* N—homozygous DNA/*2b* T—homozygous DNA (sample no 35); *3a* N—heterozygous DNA/*3b* T—MSI (sample no 12); *4a* N—heterozygous DNA/*4b* T—LOH (sample no 45)

The obtained results indicate that in all 56 studied DNA samples from NSCLC specimens, LOH/MSI frequency was present in the range of 10.00–35.71 % (mean 19.45 ± 7.14 %), depending on the marker. Representative examples of LOH/MSI derived from NSCLC cells are shown in Fig. 1.

### Total LOH/MSI frequency

The total LOH/MSI frequency (%) was evaluated separately for each marker used in the study. The highest total frequency of LOH/MSI alteration (35.71 %; 10/28 informative *loci*) was observed for the D9S1604 marker, spanning the chromosomal region 9p21.3, followed by the D11S4088 marker (30.0 %; 12/40 informative *loci*) covering the chromosomal region 11p15.5. The LOH/MSI frequencies for the markers D12S1727 (12q23.2), D1S368 (1p31.2) and D9S974 (9p21.3) were similar and assessed as follows 20 % (7/35 informative *loci*), 21.62 % (8/37 informative *loci*) and 21.88 % (7/32 informative *loci*), respectively. The lowest incidences of LOH/MSI, 12.20, 10.64 and 10 %, were observed for the three markers D7S2519, D7S2544 and D7S530 from the 7q32.2 region, respectively. Statistical analysis revealed that the observed increase in LOH/MSI frequency for D9S1604, D11S4088 and D11S1318 markers, was not statistically significant (*p* = 0.29, 0.073, 0.154 respectively; χ^2^ test). A lowest incidence in total LOH/MSI frequency among all markers was observed for D7S530, however, it was also not statistically significant (*p* = 0.0599; χ^2^ test) (see Table 4).

**Table 4:** The results of total LOH/MSI frequency evaluation in NSCLC specimens (all histological types) for 13 studied microsatellite markers, mapped to 1p, 7q, 9p, 11p, 12q and 16q (χ^2^ test)

Marker	LOH/MSI presence in marker	*p* value for the marker	Region	LOH/MSI presence in region	*p* value for the region
D1S368	8/48	0.7684	1p31.2	16/96	0.6883
D1S2137	8/48	0.7684			
D7S2519	5/51	0.3871	7q32.2	13/155	0.0623
D7S2544	5/51	0.3871			
D7S530	3/53	0.0599			
D9S974	7/49	0.9293	9p21.3	17/95	0.5097
D9S1604	10/46	0.2931			
D11S4088	12/44	0.0725	11p15.5	23/89	0.0301
D11S1318	11/45	0.1541			
D12S1041	5/51	0.3871	12q23.2	12/100	0.5144
D12S1727	7/49	0.9293			
D16S496	5/51	0.3871	16q22.1	13/99	0.6998
D16S3025	8/48	0.7684			

Focusing on a comparison of LOH/MSI total frequencies between the studied chromosomal regions, the highest total LOH/MSI frequency was observed at 9p21.3 (28.79 %) followed by 11p15.5 (28.41 %). The lowest total LOH/MSI frequency (10.94 %) was observed in the 7q32.2 region. Statistical analysis confirmed a significant increase of the LOH/MSI frequency in 11p15.5 (*p* = 0.031, χ^2^ test). The decrease of total LOH/MSI frequency in the chromosomal region 7q32.2 was not statistically significant (*p* = 0.062, χ^2^ test) (see Table 4). The results of total LOH/MSI frequency evaluation for all analyzed markers are shown in Table 4.

### Correlation of total LOH/MSI frequency with clinicopathological parameters

The comparison of the LOH/MSI frequencies in women (n = 19) and men (n = 37) depending on the studied regions, revealed the highest incidence of LOH/MSI in the 11p15.5 chromosomal region for both men and woman. The differences in LOH/MSI frequency in both men and women were observed in three studied regions: 7q32.2, 12q23.2 and 16q22.1. The LOH/MSI total incidence in these regions was found to be lower in women than in men. However, no statistically significant differences between the groups (women and men) were found in the analyzed regions: 7q32.2, 12q23.2 and 16q22.1 (*p* > 0.05, Fisher test).

The comparison of total LOH/MSI frequency between the studied chromosomal regions in all histopathological types of the NSCLC (SCC; AC; LCC) revealed a statistically significant increase of studied alteration i.e. LOH/MSI only for the LCC group in the region 12q23.2 (*p* = 0.016; *χ* ^2^ test). However the increased frequency of both markers D12S1041 and D12S1727 calculated separately was not found to be statistically significant. The highest frequencies of LOH/MSI were observed for the AC group in 7q32.2 and 11p15.5 and for the SCC group in the 9p21.3 and 11p15.5 regions, but these were not significantly higher than other findings.

The LOH/MSI frequency for all studied specimens divided into 3 groups according to pTNM staging (pT1, pT2, pT3–T4) was also investigated in every studied chromosomal region. The highest frequency of LOH/MSI in the pT1 group was observed in 7q32.2 and 11p15.5 *loci*, but the differences were not statistically significant (*p* = 0.627, 0.152 respectively; *χ* ^2^ test). Similarly, in the pT2 group, a non-significant increase of the LOH/MSI frequency was observed in 9p21.3 and 16q22.1 regions (*p* = 0.367, 0.186 respectively; *χ* ^2^ test). Interestingly, a significance increase in LOH/MSI frequency was revealed for only for one marker—D9S1604 from the 9p21.3 region (*p* = 0.027; *χ* ^2^ test). In the (pT3 + pT4) group, the rise of LOH/MSI incidence in the 11p15.5 region was observed, but with no statistical significance (*p* = 0.148; *χ* ^2^ test).

**Fig. 2:**
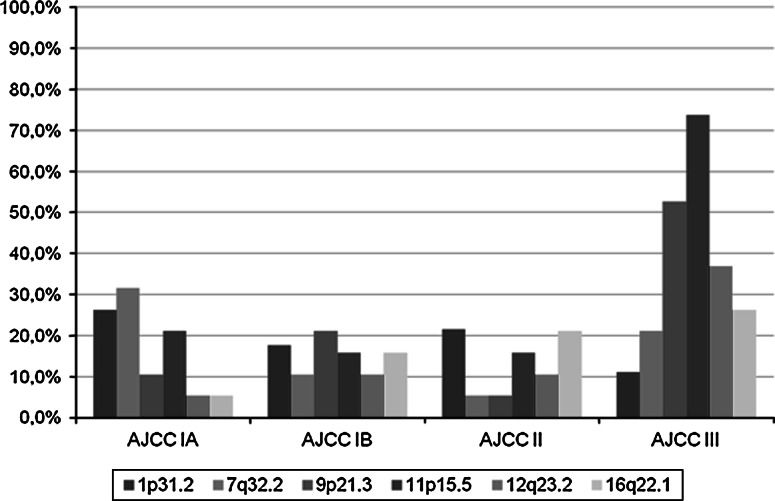
Total LOH/MSI frequency (%) in NSCLC samples in the studied chromosomal region in groups according to the AJCC classification (*AJCC IA*, *AJCC IB*, *AJCC II*, *AJCC III*)

The comparison of LOH/MSI frequency in all specimens according to the AJCC classification (AJCC IA, AJCC IB, AJCC II, AJCC III) in every chromosomal region was also investigated. Analysis revealed a statistically significant increase in LOH/MSI frequency in the AJCC III group in the 11p15.5 region (*p* = 0.007; *χ* ^2^ test), for both studied markers D11S4088 and D11S1318 (for each *p* = 0.047; *χ* ^2^ test). For the AJCC III group, a statistically significant decrease of LOH/MSI in the 7q32.2 *locus* was observed (*p* = 0.037; *χ* ^2^ test); mainly for the D7S530 marker (*p* = 0.040; *χ* ^2^ test). The total LOH/MSI frequency (%) in NSCLC samples (according to the AJCC classification) in all the studied chromosomal region are shown in Fig. 2.

The LOH/MSI frequency in all patients according to length of smoking period (less than 25 years; 25–39 years; more than 40 years) in every studied chromosomal region was also investigated. For the groups of patients who had been smoking for less than 25 years and from 25 to 39 years, the highest frequency of LOH/MSI was noticed in the 11p15.5 region, but the increase was not significant (*p* = 0.213; *χ* ^2^ test). In the age 25–39 group, the increased frequency of LOH/MSI in 11p15.5 region was statistically significant (*p* = 0.022; *χ* ^2^ test), with the marker D11S1318 being statistically significant (*p* = 0.040; *χ* ^2^test). For patients with the longest smoking history, more than 40 years, the highest LOH/MSI frequency was observed in 9p21.3 followed by 11p15.5 regions, however the increase was not significant (*p* = 0.139, 0.462; respectively; *χ* ^2^ test).

## Fal index evaluation

**Fig. 3:**
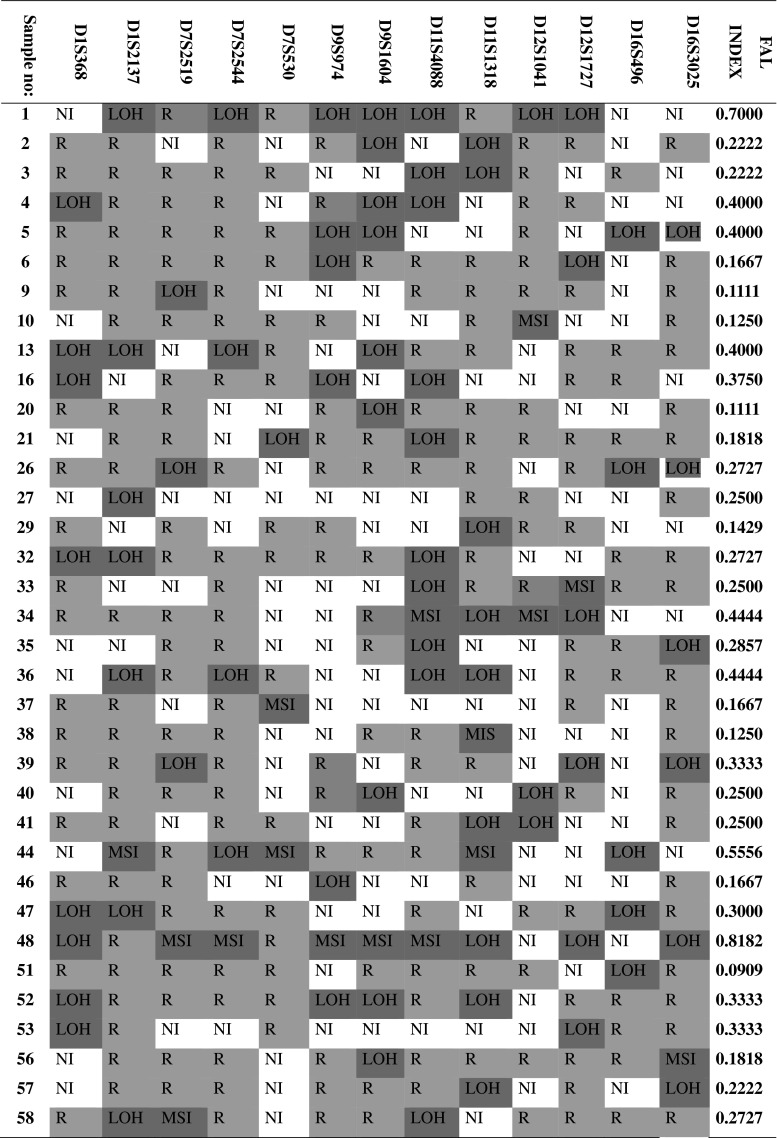
The assessed LOH/MSI frequency (FAL index) in NSCLCL specimens for 13 studied microsatellite markers, mapped to: 1p, 7q, 9p, 12q and 16q. *Note* *R* (retention of allele) informative *loci* without LOH/MSI (heterozygote); *NI* (non-informative allelotype) homozygote; *LOH* presence of loss of heterozygosity; *MSI* presence of microsatellite instability

FAL indexes were evaluated only for those NSCLC samples for which LOH/MSI was present at least in 1 microsatellite *locus*. In cases where LOH/MSI was not detected in all studied regions, the FAL value was equal to 0. The results for samples where LOH/MSI was present at least in 1 microsatellite *locus* are shown in Fig. 3.

### Correlation of FAL index with clinicopathological parameters

The FAL index values of all (56) samples were analyzed separately in relation to clinical features of patients: sex, patient’s age at time of diagnosis, as well as the histopathological characteristics of the tumours (according to TNM and AJCC classifications).

**Fig. 4:**
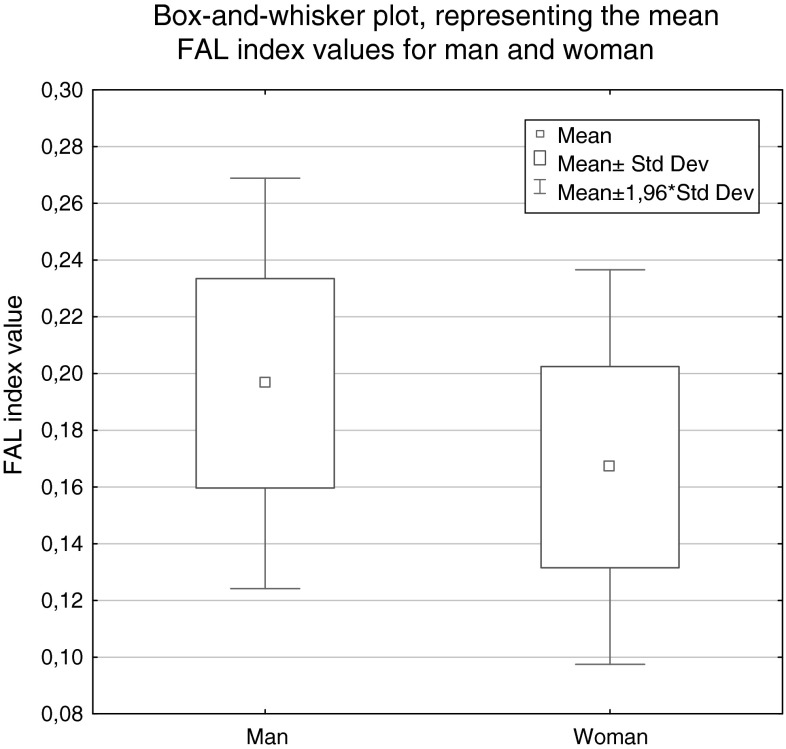
*Box*-and-*whisker plot*, representing the mean FAL index values for man and woman (*p* = 0.077; U Mann–Whitney test)

The FAL mean value in men was equal to 0.117 ± 0.143, and in woman to 0.214 ± 0.166. Statistical analysis showed no significant differences in the levels of FAL in both gender groups (*p* = 0.077; U–Man–Whitney test). The relevant graphs representing the FAL index (mean and median values) for men and woman are shown in Fig. 4.

**Fig. 5:**
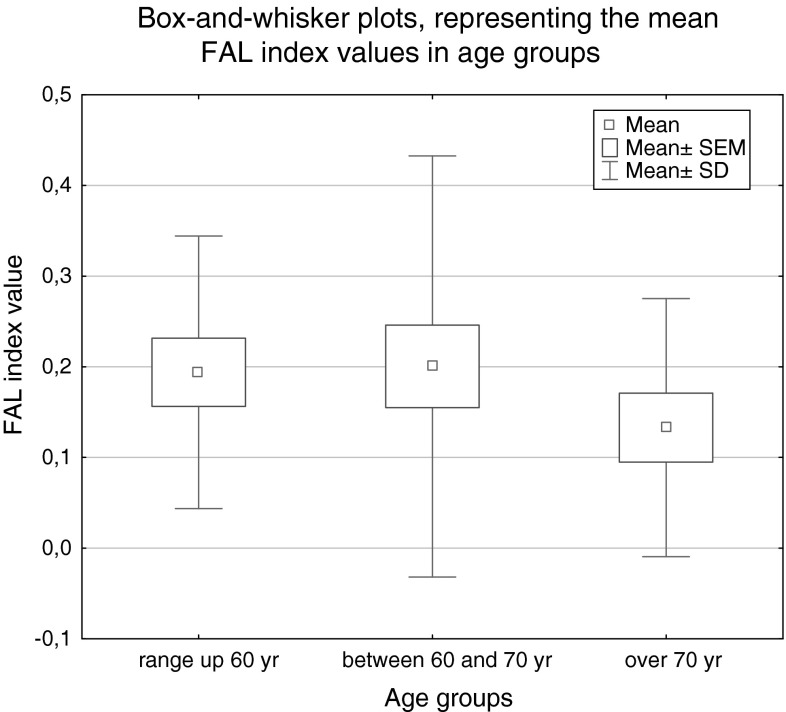
*Box*-and-*whisker plots*, representing the mean and median FAL index values for the age groups in the study (*p* = 0.590; Kruskal–Wallis test)

No statistically significant correlation was observed between FAL index levels and patient age (in the ranges up 60 years, between 60 and 70 years, and over 70 years) according to the ANOVA Kruskal–Wallis test results, *p* = 0.590 (see Fig. 5).

The differences between FAL index values in all histopathological types of NSCLC used in the study (SCC vs. AC vs. LCC) were analyzed, but they were not statistically significant (*p* = 0.217; Kruskal–Wallis test). No statistically significant difference was found in the FAL index value between the SCC subtypes: keratodes and SCC non-keratodes (*p* = 0.385; U Mann–Whitney test). Likewise no statistically significant difference was found in the FAL index value between the AC subtypes: AC mucosecretans versus AC non-mucosecretans (*p* = 0.320; U Mann–Whitney test).

The analysis of the association between the FAL index value and tumour size according to pathological staging groups (pT1, pT2, pT3–pT4) did not reveal any statistical significance (*p* = 0.185; Kruskal–Wallis test). The analysis of the association between the FAL index value and tumour stage according to the pathological AJCC staging classification groups (IA, IB, IIA, IIB, IIIA/IIB) revealed no statistical correlation (*p* = 0.435; Kruskal–Wallis test).

In our study the difference in LOH/MSI frequency was evaluated according to Imprinted region (IR) and Non-imprinted chromosomal region (NIR). Analyzed markers were divided in two groups depending on their presence in IR or NIR. Markers mapped to the 1p31.2, 7q32.2 and 11p15.5 belong to the IR, whereas 9p21.3, 12q23.2, 16q22.1 belong to the NIR. Comparison of LOH/MSI total frequency (sum of all LOH/MSI) in IR versus NIR, revealed higher frequency of LOH/MSI in NIR (0,208 ± 0,078) than in IR (0,183 ± 0,080). Statistical analysis has not confirmed the significant difference in LOH/MSI frequency in IR versus NIR (*p* = 0.520; Mann–Whitney test).

### FAL and tobacco smoking

No statistically significant correlation was revealed in the analysis of relationships between the FAL index and the smoking time period: (1) more than 40 years, (2) more than 25 years (25–39 years), (3) less than 25 years, (4) never smoked, (*p* = 0.188; Kruskal–Wallis test). However the analysis of relationships between the FAL index and the PY: up to 20; 20–29; 30–39; more than 40, revealed a statistically significant relationship (*p* = 0.024; Kruskal–Wallis test). The results are shown in Table 5.

**Table 5:** FAL index values of groups according to the time period of cigarette smoking and the Pack Years (PY)

	No. of patients	FAL mean value	SD	SEM	*p*
Smoking time period					
1. more than 40 years	20	0.23675	0.19236	0.04301	0.1881
2. more than 25 years	21	0.15095	0.15448	0.03371	
3. less than 25 years	9	0.10356	0.14191	0.04730	
4. never smoked	3	0.09100	0.15762	0.09100	
Pack years (PY)^a^					
up to 20 PY	7	0.05556	0.09623	0.03637	**0.0236**
20 to 29 PY	10	0.10136	0.11445	0.03619	
30 to 39 PY	11	0.16865	0.14015	0.04226	
More than 40 PY	22	0.25360	0.19709	0.04202	

Bold number in the last column is to highlight the calculated values of Methylation Index

^a^analyzed only in group of smokers (‘never smoking’ patients have not been included)

**Fig. 6:**
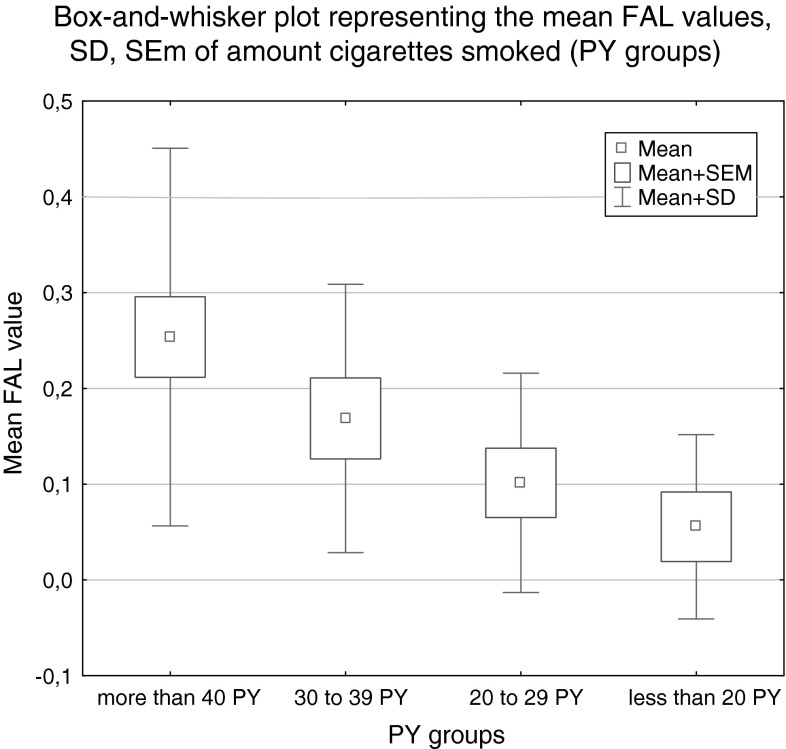
*Box-*and-*whisker plots*, representing the mean FAL index values (SEM and SD value) for study groups depending on number of cigarettes smoked in a lifetime; recalculated in PY (*p* = 0.024; Kruskal–Wallis test)

The increases in mean FAL value for the group significantly correlate with the increased number of cigarettes smoked in a lifetime (recalculated in PY), see Fig. 6.

## Discussion

In the past decade, many helpful studies focused on prognostic and predictive biological markers of NSCLC have been conducted and some of those markers are regarded as important in early cancer detection and prognosis [3]. When the heterogeneous clinical, biological and molecular nature of lung cancer is taken into consideration and different molecular changes occurring in lung epithelial cells during carcinogenesis, a study identifying molecular alterations associated with the biological behavior of the tumour is invaluable for diagnostic and therapeutic targets.

In present study, 13 microsatellite markers, related to 1p31.2, 7q32.2, 9p21.3, 11p.15, 12q23.2 and 16q22 regions, were chosen to evaluate LOH/MSI frequency. Highlighting the small amount of previously published studies on LOH/MSI frequency in 1p, 7q, 9p and 11p regions in lung cancer, our study should be acknowledged as interesting and valuable because of the analysis of many *loci* harboring genes important in cancerogenesis and located on different chromosomes [18, 20, 32]. Additionally, our evaluation of LOH/MSI presence and frequency were conducted using modern molecular methods.

In our study, LOH/MSI were observed in all chosen regions with different frequencies (10.94–28.79 %). The most frequent percentage of LOH/MSI incidence in NSCLC specimens was observed for chromosomal region 11p15.5, followed by 9p21.3 and 1p31.2. Frequent allele loss in lung cancer observed in our study within the long arm of chromosome 11 (11p), especially in the 11p15.5 region where *KCNQ1* is mapped—are similar to the results of others [20, 33]. Losses within the 11 chromosome, including the telomere region, have been documented for all histopathological types of NSCLC [21, 33]. The pivotal role of LOH/MSI incidence at chromosome 11 is largely known, especially in 11p13 region—recognized as a potential predictive marker of poor outcome in SCC [33] and in 11q23.2 [34]. The related importance of chromosome 11 alterations in lung cancer is due to the functional loss of genes mapped to the 11p15.5 chromosomal region, related to aberrant cell proliferation and/or acquired ability of clonally growth [33, 34].

According to obtained results we demonstrate that a significant increase in LOH/MSI frequency in the 11p15.5 region is associated with disease staging according to AJCC. High LOH/MSI frequency in this *locus* significantly correlates with the advance of disease—AJCC III stage (*p* < 0.005). Unfortunately, our results are not in concordance with the observations of other authors, where, despite a high frequency of LOH/MSI in the 11p15.5 *locus,* no relationship between LOH frequency and disease stage (I or II according to TNM) was found [33]. However, a relationship between the LOH incidence and survival rates, indicating LOH in 11p15.5 as being highly predictive of poor survival from NSCLC has been observed [33]. Regrettably, in the case of our patients, the survival data was not available; therefore this comparison has not been conducted. Similarly to the results of other authors [20, 33], our study also documents the universality of LOH/MSI incidence in 11p15.5 for all studied histopathological types of NSCLC (AC, SCC, LCC). Moreover, we observed that the LOH/MSI alteration was the most frequent alteration in the AC histological type, but not on significant level.

Frequent deletions in 9p were previously described according to numerical and structural changes and comparative genome hybridization (CGH) studies [5, 17, 20, 22]. Petersen et al. [22] noted that high LOH/MSI frequency was present in the loci of *p16INK4A* at 9p21, which have been confirmed in other studies [17, 22, 35]. Balsara et al. [17] demonstrated in previous study that deletion in 9p21.3 is the most frequent genetic event in NSCLC. This finding is confirmed in our study: the frequency of LOH/MSI in the 9p21.3 was the second most frequent among the analyzed regions. Mariatos et al. [35] demonstrated that allelic losses at D9S157 (9p22) occurred more frequently in the early stages of lung cancerogenesis, but we did not confirm this observation. Furthermore, in our study the highest LOH/MSI frequency was observed rather in more advanced tumours according to the TNM staging. In present study, we also analyzed the genetic instability within the 1p31.2 region that, similarly to 9p21.3, was previously examined by others [22]. Miura et al. [21] previously described the rearrangements of chromosomes 1 and recurrent loss involving 1p as well as breakpoints clustered at several chromosomal sites, including 1p13. By using comparative genomic hybridization, Petersen et al. [20, 22]. describes frequent deletions in 1p and gains in 1q21–q25—found to be associated with metastasis.

According to chromosomal aberrations in 1p31.2 (*ARHI* *locus*), they have been previously detected in a small cohort of patients. However deletion or breaks within the region 1p10–p35 were recognized [21, 32], Especially Berker-Karaüzüm et al. [32] demonstrated structural abnormalities occurring most frequently in chromosomes 1 (and chromosome 7 as well) among all analyzed regions in his study. In our study, the mean frequency of LOH/MSI in studied cohort in 1p31.2 region was average (16.67 %) as compared to the frequencies found by other [21, 32], which does not confirm significance of LOH/MSI in this region. In our study, the LOH/MSI presence in this region is documented in all studied histological types, with the most frequent incidence of LOH/MSI noted in the SCC type, which does not confirm the findings of Berker-Karaüzüm [32].

Another analyzed chromosomal region in our study, 16q22.1, hasn’t been studied in LOH analysis in lung cancerogenesis. However, in other human cancers, i.e. breast and thyroid, genetic instability was often detected in this region [26, 36]. *E*-*cadherin* is one of the genes within this *locus*—its protein product plays a crucial role in epithelial cell–cell adhesion, intercellular adhesion, and can influence the cell transformation. Downing et al. [36] focused on this *locus* in breast cancer and revealed that the increased genetic instability in this region in younger patients (less than 45 yr) is related to worse prognosis, and a more aggressive tumour. In our study, no significant differences in LOH/MSI frequency in 16q22.1 were observed between the age groups. Bremnes et al. [10] showed that the reduced membranous *E*-*cadherin* expression was related to LCC histology, dedifferentiation, local invasion, regional metastasis, and significantly reduced survival. In our study, not only an elevated LOH/MSI frequency was found in the SCC histological type, but also the absence of LOH/MSI in LCC specimens was confirmed. It seems to be promising observation in relation to the expression gene profile, which is worth evaluation. In our study, frequent LOH/MSI in this region was observed in patients with a long smoking history of more than 40 years, and so may be associated with the length of tobacco addiction. However no data is available from other studies concerning the influence of tobacco.

The LOH/MSI frequency was quite low in the two regions analyzed in our study: i.e. in 12q23.2 and 7q32.2. Gains in 12q have been previously detected, however loss of heterozygosity in this region hasn’t been frequently observed. The chromosomal breakpoint on the long arm of the 12 chromosome (12q14) were identified, but in lung cancer cell lines rather than in a cohort of patients. Ding et al. [29] observed large deletion (12q15–q21) occurring at the later stages of NSCLC progression (*p* < 0.05). An interesting finding in our study was the elevated LOH/MSI frequency in 12q23.2 in the LCC histological type, and this finding was statically significant (*p* < 0.05). However the study group was not numerous, thus the conclusions might not be valid. We did not find other reports on LOH/MSI frequency with regard to NSCLC histological type in this region. Ding et al. [29] showed the association between the presence of LOH in the *loci* of microsatellite markers (D12S2196, D12S398, D12S326, and D12S106) with cancerogenesis in patients with no smoking history. In our study, no association could be made with the smoking history due to the very small group of non-smokers in the studied cohort: 3/56 patients. According to our findings, the incidence of LOH/MSI within the studied region was not elevated in long-smoking patients, more than 40 years, nor in the 25–39 age group. We did not prove the relationship between the tobacco addiction length and presence of LOH/MSI in analyzed *loci*. However, in our study we documented the relationship between the mean FAL value index with cigarette consumption recalculated in PY (*p* < 0.05). The amount of cigarettes smoked in a lifetime (in PY) seems to have impact on the increase in genetic instability, i.e. LOH and MSI. This finding confirms the results of others [19, 37]. The analysis of the relationship between genetic instabilities i.e. LOH/MSI and tobacco addiction revealed more significant impact of amount of cigarettes smoked in lifetime (PY number) than the overall smoking period. Interestingly enough, chromosomal gains in 3q, 8q in LCC are thought to be associated with smoking-induced lung cancer [24]. Our observations, i.e. the high incidence of LOH/MSI observed in LCC patients along with their extended smoking history, seem to confirm these findings.

Another region analyzed in our study was the 7q32.2 harboring the *MEST* gene. The instability on chromosome 7 has been previously studied, with the most studied alteration, LOH in the 7p12 region—*EGFR* oncogene *locus* [3, 23]. The existences of „hot spots” for cytogenetic change involving specific regions on chromosome 7, other than 7p12, were also described [21]. A chromosomal aberration in 7q32 (*MEST* *locus*) has been previously detected in a small cohort of patients by Berker-Karaüzüm et al. [32] who demonstrated LOHs in 8/30 cases. Our study demonstrates the presence of LOH in the 7q32.2, but frequency of this genetic alteration was the lowest among all the studied regions. The mean FAL value in this region, as compared to other studied *loci* revealed tendency to decrease (*p* > 0.05). It should be stressed that our results did not confirm high LOH frequency within chromosome 7 (7p14.3, 7p22.2, and 7q24.3) observed by Tseng et al. [14]. On the other hand *loci* taken into consideration in our study were different [14]. Presence of LOH in Tseng et al. [14] study revealed the correlation with cancer progression, and/or poor prognosis. Our results—however based on different markers—did not completely confirm this observation. In our study, the correlation between LOH/MSI frequency and the advanced stage of lung cancer (AJCC III), in the 7q32.2 was found. In the MEST *locus* genetic alteration other than LOH/MSI were observed e.g. in lung cancer cell lines. Nakanishi H et al. [38] noted that frequent loss of imprinting (LOI) of paternally imprinted PEG1/MEST led to expression changes, but no study was conducted to confirm whether the LOH in this region could cause the LOI.

Molecular analyses of chromosomal aberrations, like LOH/MSI alteration, in lung cancers have allowed selection of chromosomal regions which are highly altered in lung cancers, indicating possible distant carcinogenesis pathways in lung. Also, molecular characteristic of histological types such as adenocarcinoma histological type may develop which improves the cytological examination. In our study, we did not completely confirm the previously published data about the high frequency of LOH/MSI alteration in different chromosomal *loci* analyzed in NSCLC specimens. In fact, accurate comparison of our results with those of others seem to be difficult in a view of using a large group of analyzed *loci* (13 markers located in 7 regions) in our study. This approach can not to be fully compared with findings from studies based on a small number of markers located in the same chromosomal region, and/or different (especially smaller) cohort of patients.

Despite of many differences in our results in comparison with other authors it should be noted that high frequency of instability in 11p confirmed in our study in correlation with more advanced tumor (according to the AJCC Classification) may have important value for investigation of new informative prognostic marker in the future. Additionally, the LOH/MSI presence observed by us in 7q32.2 region (despite of low frequency) and confirmed correlation with advanced stage cancer (AJCC III) may include significant charge in establishing diagnostic and/or prognostic markers.

Finally, calculation of FAL index value from several *loci* can be useful as universal marker of LOH/MSI alteration in lung cancer and also as an indicator of their correlation with same factors important in lung carcinogenesis, such as tobacco smoking.
